# Rotavirus and Serotonin Cross-Talk in Diarrhoea

**DOI:** 10.1371/journal.pone.0159660

**Published:** 2016-07-26

**Authors:** Sonja Bialowas, Marie Hagbom, Johan Nordgren, Thommie Karlsson, Sumit Sharma, Karl-Eric Magnusson, Lennart Svensson

**Affiliations:** 1 Department of Clinical and Experimental Medicine, Division of Molecular Virology, Linköping University, Linköping, Sweden; 2 Department of Clinical and Experimental Medicine, Division of Medical Microbiology, Linköping University, Linköping, Sweden; Karolinska Institutet, SWEDEN

## Abstract

Rotavirus (RV) has been shown to infect and stimulate secretion of serotonin from human enterochromaffin (EC) cells and to infect EC cells in the small intestine of mice. It remains to identify which intracellularly expressed viral protein(s) is responsible for this novel property and to further establish the clinical role of serotonin in RV infection. First, we found that siRNA specifically silencing NSP4 (siRNA^NSP4^) significantly attenuated secretion of serotonin from Rhesus rotavirus (RRV) infected EC tumor cells compared to siRNA^VP4^, siRNA^VP6^ and siRNA^VP7^. Second, intracellular calcium mobilization and diarrhoeal capacity from virulent and avirulent porcine viruses correlated with the capacity to release serotonin from EC tumor cells. Third, following administration of serotonin, all (10/10) infants, but no (0/8) adult mice, responded with diarrhoea. Finally, blocking of serotonin receptors using Ondansetron significantly attenuated murine RV (strain EDIM) diarrhoea in infant mice (2.9 *vs* 4.5 days). Ondansetron-treated mice (n = 11) had significantly (p < 0.05) less diarrhoea, lower diarrhoea severity score and lower total diarrhoea output as compared to mock-treated mice (n = 9). Similarly, Ondansetron-treated mice had better weight gain than mock-treated animals (p < 0.05). A most surprising finding was that the serotonin receptor antagonist significantly (p < 0.05) also attenuated total viral shedding. In summary, we show that intracellularly expressed NSP4 stimulates release of serotonin from human EC tumor cells and that serotonin participates in RV diarrhoea, which can be attenuated by Ondansetron.

## Introduction

Rotavirus (RV) is the leading cause of acute gastroenteritis in infants and young children worldwide and associated with significant mortality [1]. Most deaths result from an excessive loss of fluids and electrolytes through vomiting and diarrhoea. Despite its large clinical importance and years of research, the knowledge on the pathophysiological mechanisms that underpin this life-threatening disease remains limited. Several mechanisms have been proposed to account for the watery diarrhoea associated with RV infection. These include imbalance in osmosis following virus-induced loss of epithelial absorptive functions, effects of the virus-encoded enterotoxin NSP4 and/or an active role of the enteric nervous system (ENS) and neurotransmitters [2–7]. Moreover, a RV-infection has been shown to stimulate vagal afferent nerves to the *nucleus tractus solitarii* (NTS) in the brain stem, a structure in the vomiting center [3].

RV has been shown to infect mature enterocytes in the tip of the villi of the small intestine [6, 8]. Recently it has also been shown that RV can infect enterochromaffin (EC) cells [3]. The EC cells are the largest enteroendocrine cell population in the small intestine. They are characterized by their synthesis and release of the 5-hydroxytryptamine (5-HT, serotonin)[9, 10]. EC cells can “taste” and “sense” the luminal contents and release mediators such as serotonin to activate ENS, as well as extrinsic vagal afferents to the brain. They are the only neuroendocrine cells in the human body that actively synthesize serotonin in the digestive tract and small intestine while other cell types like epithelial cells in the lining of the intestines only store to degrade serotonin produced by EC cells [11, 12]. EC cells are strategically positioned in the intestinal mucosa to release mediators of endocrine signalling from the basolateral surface activating afferent neuron endings within the *lamina propria* [13, 14]. Upon stimulation by several factors, e.g. hyperosmolarity, carbohydrates, mechanical distortion of the mucosa, cytostatic drugs and toxins like cholera toxin [14, 15], EC cells mobilize intracellular Ca^2+^ followed by release of serotonin [10]. Serotonin is involved in the regulation of gut motility, intestinal secretion, blood flow, several gastrointestinal (GI) disorders [16–20], illness and acute gastroenteritis [21, 22] and *Staphylococcal* enterotoxin-induced vomiting [23]. Serotonin has also been shown to affect immune and inflammatory response by regulating cytokine levels [24, 25].

Synthesis of serotonin in EC cells is regulated by the rate-limiting enzyme tryptophan hydroxylase (TPH) localized in the pineal gland and gut intestinal EC cells [26–28]. To prevent receptor desensitization by excess of serotonin, cells utilize the serotonin reuptake transporter (SERT) to transport serotonin across the cell membrane for internal storage and terminating serotonergic signalling [29–31]. This sodium-and chloride-coupled transporter is localized on both sides of the cell membrane [32] along the human intestine with the highest expression in the ileum [31] thereby it can play a key role in the regulation of serotonin content and availability of serotonin along the GI tract [33]. Moreover, all epithelial cells in the intestine that express SERT can take up extracellular serotonin to control extracellular serotonin levels to avoid desensitization of the 5-HT receptors [16, 29, 34, 35]. Two clinical studies have indeed reported a reduction in serotonin reuptake due to reduced levels of SERT in patients with IBS and ulcerative colitis [16, 36]. Furthermore, decreased SERT expression was observed in the small intestine of mice infected with enteropathogenic *E*. *coli* (EPEC) [37].

We have previously shown that extracellularly added NSP4 can stimulate secretion of serotonin from human EC tumor cells [2, 3]. While RV can infect murine EC tumor cells *in vivo* and human EC tumor cells *ex vivo* and stimulate secretion of serotonin in a dose-and time-dependent manner [3], it remains to be determined if an intracellularly expressed viral protein is responsible for this unique neurotransmitter-stimulation property.

The serotonin receptor antagonist Ondansetron is used to attenuate illness in children with acute gastroenteritis [21, 22] and diarrhoea in patients with IBS [38, 39]. Furthermore, it has documented obstipation effects [40, 41]. While administration of commercially available Ondansetron such as Zofran^©^ has documented beneficial effects on various gastrointestinal illnesses there is no information on whether RV-induced diarrhoea can be attenuated by Ondansetron. This question was also addressed in this study.

Our siRNA silencing experiments, showed that intracellularly expressed NSP4 stimulated serotonin secretion from human EC tumor cells and that its effect accounted for most of the serotonin release as compared to other investigated viral proteins. Moreover, calcium mobilization and diarrhoea capacity from virulent and avirulent porcine (strain OSU) viruses [42] correlated with release of serotonin from EC tumor cells. Importantly, blocking of serotonin receptors by Ondansetron attenuated murine rotavirus (strain EDIM) diarrhoea in infant mice and significantly (p < 0.05) reduced the total viral shedding in adult mice. The objectives with this study were to: (i) identify the intracellularly expressed viral protein with serotonin-stimulating-capacity and to (ii) further investigate the cross-talk between RV and serotonin in diarrhoea. We found that NSP4 carries serotonin-stimulating properties and that Ondansetron reduced RV-induced diarrhoea. The importance of these observations in the context of disease mechanisms is discussed.

## Material and Methods

### Cells, viruses and antibodies

The rhesus monkey kidney epithelial cell line MA104 (ATCC CRL 23781) was cultured in Eagle’s minimal essential medium (Eagles MEM) (D6046, Sigma-Aldrich, USA) supplemented with 10% fetal bovine serum (FBS), 2 mM L-glutamine (M11-004, GE Healthcare, Austria), 0.02 mg/mL gentamycin (P11-004, GE Healthcare), 1X MEM non-essential amino acids (M11-003, A&E Scientific (PAA), Belgium) and 1 mM sodium pyruvate (SH30239.01, Thermo Scientific, USA) at 37°C in 5% CO_2_. The human EC cell line (GOT1), a tumour derived enterochromaffin cell line arising from a midgut carcinoid tumour [43] was cultivated in RPMI 1640 medium (R0883, Sigma-Aldrich) supplemented with 10% FBS, 1X MEM non-essential amino acids, 0.02 mg/mL gentamycin and 5 mM L-glutamine. Cells were tested to be free from mycoplasma using MycoAlert^TM^ mycoplasma detection kit. Rhesus rotavirus (RRV) and wild-type murine RV (EDIM strain) were used in most experiments. The OSU virulent (OSU-v) and attenuated (OSU-a) RV were kindly provided by Dr. Linda Saif (Ohio State University, Ohio, USA).

Monoclonal antibodies against VP7 (M60) and VP4 (HS1) were kindly provided by Dr H. B. Greenberg, Stanford University, USA. The monoclonal antibody against serotonin was purchased from Dako (M758, Dako Cytomation, Denmark). Rabbit polyclonal antibodies included anti-NSP4 [44] and anti-VP6 (K224). Secondary antibodies for immunofluorescence microscopy were rhodamine-labelled goat anti-rabbit IgG (111-025-045, Jackson ImmunoResearch, USA) and fluorescein (FITC)-labelled goat anti-mouse IgG (115-095-003, Jackson ImmunoResearch). Horseradish peroxidase (HRP)-conjugated goat anti-rabbit (170–6515, Bio-Rad, USA) and goat anti-mouse (170–1011, Bio-Rad) were used as secondary antibodies while performing western blot analysis and immunoperoxidase staining.

### Animals

BALB/c mice (B&K Laboratories, Sollentuna, Stockholm, Sweden) were used and housed in standard cages with free access to food and water. Pregnant females were transferred to individual cages 1 week before the expected day of birth, and offspring remained with their mother during the experimental period. Animal experiments were approved by Stockholm Norra Djurförsöksetiska nämnd (the local Ethical Committee), Stockholm, Sweden; Approval No: N289/09, N291/010 and N256/14.

### Definition of diarrhoea

Mice were examined once a day for signs of diarrhoea, as defined by liquid yellow stools induced by gentle abdominal palpation as described previously [45]. Diarrhoea was scored 1–3 with 3 being the most severe and verified by two observers independently. Individual mice were followed each day after inoculation and the total number of days with diarrhoea (NDD) and weight of the mice were noted. The daily percentage of mice with diarrhoea was calculated by dividing the number of mice with diarrhoea by the number of mice in the group.

### Serotonin administration and diarrhoea

EC tumor cells (1 x10^6^ cells/well) cultured in a 6-well plate were infected with RRV at a MOI of 1. At 1 hours post infection (h p.i) cells were washed twice and incubated with serum-free Eagles-MEM. At 10 h p.i cell media was collected, centrifuged at 580 x g to remove cell debris and serotonin concentration determined in the supernatant. Eight 5–7 days old BALB/c mice were given intra-peritoneally 50 μL cell medium containing 0.04 μg (7.4 μg/kg) of serotonin and 8 mice were given 2 x 0.04 μg. As control, 7 mice were administered supernatant from infected MA104 cells. Infant mice (n = 10) also received approximately 700-fold higher dose (5 mg/kg) of serotonin (H9523, Sigma-Aldrich). Adult BALB/c mice (n = 8) were intra-peritoneally administered 5 mg/kg of serotonin and observed for diarrhoea every 30 min for 5 h in total.

### Rotavirus infection and treatment studies

Five-to seven days old and adult BALB/c mice were orally infected with 100DD_50_ (diarrhoeal doses) of murine rotavirus (strain EDIM) 10 μL/animal as previously described [3]. Mice were sacrificed 24 and 48 h p.i and duodenum, jejunum and ileum were collected and stored at -20°C until further analyse of TPH1 and SERT mRNA levels. In another set of experiments EDIM infected infant mice where treated twice a day (10 μL orally) with Ondansetron (n = 11) (O3639, Sigma-Aldrich) (5 mg/kg) or 0.9% saline (n = 9) (mock), until 144 h p.i. As control, Ondansetron was in parallel given to uninfected infants (n = 4), in equal concentration and time points. To investigate the effect on viral shedding, independently of diarrhoea, infected adult mice were treated orally with Ondansetron (5 mg/kg) (n = 10) or mock-treated (n = 10) twice a day, starting 4 h p.i until 144 h p.i. For analysis of viral load, fecal pellets were collected at the same time point once a day from each individual animal. The fecal pellet was stored at -20°C until analysis.

### siRNA transfection

siRNA sequences targeting the viral genes encoding NSP4, VP6, VP7 and VP4, as previously reported [46–49], and siGENOME Non-Targeting siRNA (Nt-siRNA) were synthesized by Thermo Scientific. MA104 and EC tumor cells were transfected with siRNA as previously described by Cuadras et al. [49]. Incubation time-points and siRNA concentrations differed, depending on each siRNA and cell line (Table 1). Briefly, 1 x 10^6^ EC tumor cells/ well and 2.5 x 10^5^ MA104 cells were seeded in 6-well respectively 48-well plates (Nunclon Delta Si, Denmark). Transfection with a mixture of siRNA, Lipofectamine 2000 (Invitrogen, USA) and Opti-MEM (Invitrogen) was done at 80% confluence of the cells. After 4 h or 7 h, the transfection mixture was removed and replaced with MEM containing 10% FBS. Both cell types were then incubated for another 20–24 h.

**Table 1:** siRNA concentration, sequence and transfection incubation time-points in MA104 and EC tumor cells for NSP4, VP6, VP7 and VP4.

Gene product	siRNA sequence	siRNA*	Incubation time (h)
**NSP4**	*AAACGUCAAAGUGUUCAUAUA*	*143*^*a*^*/286*^*b*^	*4* ^*a*^*/7h* ^*b*^
**VP6**	*UGGAACGAUAAUAGCCAGAAA*	*600* ^*a*^ */600* ^*b*^	*4* ^*a*^*/7h* ^*b*^
**VP7**	*AAGUCGCUACAGCUGAAAAAC*	*600* ^*a*^ */600* ^*b*^	*4* ^*a*^*/7h* ^*b*^
**VP4**	*UCUAGGUCCUUUUGCUCAAUU*	*286* ^*a*^ */286* ^*b*^	*4* ^*a*^*/7h* ^*b*^

All siRNA were synthesized with 3´-UU overhangs.

^a^ MA104 cells

^b^ EC tumor cells

* **(pmol/mL)**

### Rotavirus infection

RRV was cultivated and its titer determined as previously described [45]. Prior to the infection, RRV was activated with 10 μg/mL trypsin, for 60 min at 37°C (T8353, bovine pancreas, type III, Sigma-Aldrich) as described [45] and then the cells were infected for 1 h at an MOI of 0.5. Cells were then washed twice with serum-free Eagles-MEM and further incubated for 7 h in serum-free Eagles-MEM (supplemented with 2 mM L-glutamine and 0.02 mg/mL gentamycin). Cells were washed, fresh media added and after 1 h supernatant was collected for serotonin estimation.

### Immunofluorescence

MA104 and EC tumor cells were transfected with respective siRNA and then infected with RRV as described above. At 7 h p.i cells were trypsinized and fixed on microscope slides with ice-cold aceton (A/0520/PB17, Fisher Chemical, USA). They were further prepared for immunohistochemistry. Briefly, specimens were blocked with 1% bovine serum albumin (BSA) in PBS for 30 minutes at room temperature (R.T). Respective primary antibody was added to the cells and incubated in a humid chamber for 1 h at R.T. Following 3x washing with PBS, a secondary rhodamine goat anti-rabbit or fluorescein (FITC) goat anti-mouse IgG was added and incubated for 1 h in a humid chamber at R.T. Following 3x washes of specimens, nuclear counterstaining was performed with 300 nM DAPI (D1306, Invitrogen) diluted in PBS for 10 min. After 3 more washes with PBS specimens were mounted with fluorescence mounting media (S3023, Dako Cytomation). Fluorescence was examined by fluorescence microscopy (Nikon Eclipse E600) and images were captured with a digital camera (Nikon DXM 1200F, Japan).

Double staining of serotonin and VP6 was performed on both RRV-infected and uninfected EC tumor cells, as previously described [3]. Briefly, infected cells were trypsinized 18 h p.i, dropped on microscope slides and fixed with 4% paraformaldehyde (PFA) (02176, Histolab, Sweden) in PBS on microscope slides overnight at 4°C. Cells were washed with PBS and then treated with 0.1% Triton X-100 in PBS for 15 min at R.T. Cells were washed with PBS and incubated for 1h with mouse anti-serotonin (M0758, Dako, Denmark) (diluted 1/50 in PBS) in a humidified chamber at R.T, washed 3x with PBS and incubated for another 1h with rabbit RV antiserum (K224) diluted 1/500. After 3 washes, a mix of rhodamine goat anti-rabbit IgG diluted 1/400 and fluorescein (FITC) goat anti-mouse IgG diluted in 1/200 was added to the cells and incubated for 1 h at R.T in a humidified chamber. Following 3 washes, 300 nM DAPI in PBS was added to the cells and incubated for another 10 min. After 3 washes, specimens were mounted with fluorescence mounting media (S3023, Dako Cytomation) and examined by confocal microscopy (Axiovert 200 M, Zeiss, Jena, Germany)

### Confocal microscopy

Images were taken with Axiovert 200 M microscope stage equipped with a mercury short-arc lamp (HXP120c; Carl Zeiss), a structured illumination-aperture correlation unit (VivaTome, Zeiss) with FITC (ex; 494/20–25, em; 536/40–25), and TexasRed (ex; 575/25–25, em; 628/40–25) activations in combination with a triple band dichroic mirror (436/514/604). A 40x (NA 1.3; Carl Zeiss) objective was used for all images. The detector for this system was an Axiocam MRm CCD camera with a pixel size of 6.45×6.45 μm.

### Western blot

Semi-confluent MA104 and EC cell monolayers in 6-well plates were transfected and infected as described above. After collecting media 7 h p.i., lysis buffer containing 2% Triton X-100, 1% SDS, 0.15 M NaCl and 0.1M Tris-HCl was added to each well and freeze-thawed 3–4 times. Cell lysates were then centrifuged at 10 000 x g for 10 min and the supernatant collected and boiled for 10 min at 95°C in loading buffer (5% 2-Mercaptoethanol, 161–0710 Bio-Rad in Laemmli Sample Buffer, 161–0737 Bio-Rad) before separation with 10% polyacrylamide gel electrophoresis (PAGE). The proteins were stained with Coomassie Brilliant Blue and the relative protein concentration determined. Equal relative concentration of each sample was separated on a PAGE gel and transferred (western blot assay) to a polyvinylidene difluoride (PVDF) membrane at 375 mA for 60 min. The membrane was blocked with 3% BSA in PBS-T buffer (PBS containing 0.05% Tween) for 1 h. Antibodies specific to NSP4 (dilution 1/400 in PBS-T with 1% BSA), VP4 (dilution 1/300), VP6 (dilution 1/500) and VP7 (dilution 1/30) were added and incubated for 2 h at R.T. The membranes were washed 4x with PBS-T. HRP goat anti-rabbit (170–6515, Bio-Rad) or goat anti-mouse (172–1011, Bio-Rad) was used as secondary antibody at dilutions of 1/10000 and the membrane was incubated for 90 minutes. After washing, the reaction was developed with Bio-Rad Immun-Star HRP substrate (170–5041, Bio-Rad) and the bands were visualised with Molecular Imager® ChemiDoc™ XRS (Bio-Rad) together with Quantity One® 1-D analysis software (Bio-Rad).

### Intracellular calcium clamping

To analyse the possible connection between intracellular calcium and release of serotonin from EC tumor cells, BAPTA/AM (B-1205, Molecular Probes Thermo Fisher, USA) (C_34_H_40_N_2_O_18_) a calcium-specific chelator was used to block intracellular calcium transients in EC tumor cells. EC tumor cells were infected with RRV for 1 h at an MOI of 0.5. Pre-toxicity testing of BABTA/AM revealed that 25 μM was the most appropriate concentration for the EC tumor cells. BAPTA/AM was added to RRV-infected cells in fresh media at 3 h p.i. The cell supernatants were collected 7 h p.i and stored at -80°C until analysis of serotonin by ELISA. Serotonin release from RRV-infected cells, with and without BAPTA/AM was compared. Uninfected cells with or without BAPTA/AM and infected cells without BAPTA/AM were used as controls.

### Serotonin ELISA

A commercial serotonin ELISA kit was used (RE59121, IBL International, Germany) to determine serotonin concentration according to the manufacturers instructions.

### RNA extraction and reverse transcription from the small intestine

1 cm segments from duodenum, jejunum and ileum were collected from each mouse intestine of respective groups (uninfected, 24 h p.i and 48 h p.i). RNA was subsequently extracted from the tissue with RNeasy Plus Mini Kit (74134, Qiagen, Germany) according to the manufacturer’s instructions. Absence of cDNA was confirmed by running glyceraldehyde-3-phosphate dehydrogenase *(GAPDH)* specific real-time PCR on the RNA extract. Following determination of RNA concentration (NanoDrop ND-1000 Spectrophotometer, Saveen Werner, Life Science, Sweden), reverse transcription was performed with the AffinityScript Multiple Temperature cDNA Synthesis kit (200436, Agilent, Sweden) according to the manufacturer’s instructions. RNA in amounts of 1–0.5 μg was used for reverse transcription of jejunum, ileum and the duodenum samples. RNA was stored at -80°C until analysis by quantitative real-time PCR.

### Real-time quantitative PCR for SERT, TPH1 and GAPDH

*SERT*, *TPH1* and *GAPDH* primer sequences were obtained from previous studies [50, 51]. cDNA were quantified by SYBR Green based real-time PCR, with *GAPDH* as a reference gene. The real-time PCR reaction was performed with an ABI 7500 (Applied Biosystems, USA) with the following cycling conditions. First denaturation was done for 10 min at 95°C followed by 40 cycles of 15 s at 95°C, 1 min at 60°C, followed by melting curve analysis. Negative controls for RNA extraction, cDNA synthesis and non-template control (NTC) were included in each run. Results were analysed using the ΔΔCt method and presented as normalized data.

### Extraction of viral RNA in faeces

Fecal pellets were weighed and dissolved in 400 μL of PBS, stored over night at 4°C and subsequently vortexed. Samples were centrifuged at 13 000 x g for 10 min and the supernatant transferred to a new tube for extraction of viral RNA, using Magattract Viral RNA M48 kit (955235, Qiagen), according to the manufacturer’s instructions. RNA samples were stored at -80°C until reverse transcription and analysis by quantitative real-time PCR.

### Quantitative PCR for detection of Rhesus rotavirus

MA104 cells grown in 24-well plates were infected with trypsin-activated Rhesus rotavirus (RRV) at a multiplicity of infection of 0.1 [52]. After 1h of infection, serum-free Eagles MEM containing 10 μM Ondansetron was added. For controls, only media was used. At 48 h p.i RNA was extracted from cell supernatant and cell-lysates and analysed with a one-step quantitative RV PCR [53] with external plasmid standard.

### Detection of RRV-infected cells

The number of infected cells in Ondansetron-treated (10 μM) and non-treated cells at 16 h p.i was determined by immunoperoxidase staining using an anti-NSP4 polyclonal antibody (1:400). Briefly, trypsin-activated RRV (MOI 0.1) was added to confluent MA104 cells in 96-well plate. After 1 h serum-free Eagles MEM containing 10 μM Ondansetron was added, while for controls only media was used and the plate was incubated at 37°C with 5% CO_2_. After 16 hours of infection, cells were fixed with 4% paraformaldehyde in PBS at R.T for 2 hours. After being washed with PBS, cells were permeabilized by incubation with 1% Triton X-100 in PBS for 10 min at R.T. Rotavirus-infected cells were identified by immunoperoxidase staining essentially as described [45] using a rabbit anti NSP4 antibody [44] and peroxidase-labelled goat anti-rabbit (Bio-Rad) diluted 1:1000. The reaction was developed with aminoethylcarbazole (1 mg/ml) in 0.1 M acetate buffer (pH 5.2) and stained cells (3 independent wells) were counted under a light microscope.

### Determination of RRV infectious titre

For determining viral titres confluent MA104 cells in 24-well plates were infected with trypsin-activated RRV at MOI of 1. After 1 hour of infection, wells in duplicate were treated with either Ondansetron (10 μM) or mock-treated and incubated for 16 hours at 37°C with 5% CO_2_. Next the cells were freeze-thawed 3 times and the virus lysate was diluted in 10-fold dilutions and added in triplicates onto confluent MA104 cells in a 96-well plate. Following 16 hours of infection cells were fixed with 4% paraformaldehyde in PBS and virus titre determined as previously described [52] and expressed as pfu/ml.

### Reverse transcription of fecal RNA

RNA (28 μL) was mixed with 2.5 μg of random hexadeoxynucleotides (pd[N[12]_6_) primer (27-7858-03, GE Healthcare), denatured at 97°C for 5 min and chilled on ice for 2 min. The suspension was then added to 1 illustra^TM^ Ready-To-Go^TM^ RT-PCR Bead (27-9259-01, GE Healthcare) with RNase-free water to a final volume of 50 μL. The RT reaction was performed for 40 min at 42°C for cDNA synthesis.

### Quantitative real-time PCR for determination of viral load in faeces

Real-time PCR was performed in duplicate. Twenty μL mixtures containing 10 μL of Power SYBR Green PCR Master mix (Applied Biosystems) and 50nM of forward and reverse primers were analysed on a 7500 fast real-time PCR system (Applied Biosystems) with following cycling conditions: 95°C for 10 min followed by 40 cycles of 95°C for 15s and 60°C for 1 min. Melting curve analysis was performed after each run for specificity analysis. An EDIM positive stool sample in a 1:10 dilution series was used as standard curve, with the lowest detected dilution arbitrarily set at 30 genome copies. If RV was undetected a value of 15 genes per PCR reaction was set. Primers used were taken from a previous study [54].

### OSU-v and OSU-a infection

The virulent (OSU-v) and attenuated (OSU-a) porcine viruses have been previously characterized [42]. The OSU-v strain, being a fecal sample, was passaged 7x (P7) in MA104 cells as toxic effect of faeces was observed on the EC tumor cells. Viral titration was performed on EC tumor cells (S1 Fig). For serotonin stimulation experiments, both viruses were activated with trypsin (10 μg/mL) for 1 h at 37°C and diluted to a MOI of 1 and added to the EC tumor cells. After 1 h and 7 h p.i supernatant were collected and analysed for serotonin concentration.

To exclude the possibility that the *NSP4* gene of OSU-v strain had mutated during the 7 passages in MA104 cells, sequencing of the NSP4 gene was performed. Briefly, RNA extraction was done from 140 μL of the OSU-v P7 strain using QIAamp Viral RNA Mini Kit (Qiagen, Germany) according the manufacturer’s instructions. Twenty-eight μL of RNA was subsequently mixed with 50 pmol of random hexadeoxynucleotides [pd(N)_6_], denatured at 97°C for 5 min, and chilled on ice for 2 min, followed by addition of one illustra^TM^ Ready-To-Go^TM^ RT-PCR Beads (GE Healthcare) and RNase-free water to a final volume of 50 μL. The RT reaction was carried out for 42 min at 42°C to produce the cDNA. The NSP4 gene was amplified using GEN_NSP4F 5’- GGC TTT TAA AAG TTC TGT TCC -3’ and GEN_NSP4R 5’- GGW YAC RYT AAG ACC RTT CC -3’ as previously described [55] and the (750 bp) PCR product was sequenced.

### Statistical analyses

The Mann-Whitney U-test was used to analyse differences in the quantification of serotonin levels and the mean fluorescent intensity from the serotonin expression studies. Differences in gene expression between uninfected and RV-infected mice were analysed with Kruskal-Wallis and Bonferroni’s multiple comparison test. Two-tailed tests were used and p < 0.05 was considered statistically significant. Unpaired t-tests were used to analyse data from OSU and the Ondansetron-treated infant mice studies. Mann-Whitney test was used to analyse data from the viral shedding and Ondansetron-treated adult mice. The statistical analysis was performed with GraphPad prism (GraphPad Prism 5.0a Macintosh Version by Software MacKiev © Copyright GraphPad Software, Inc. 1994–2008).

## Results

### Calcium mobilization and diarrhoea properties of virulent and attenuated OSU virus strains correlate with the release of serotonin from human EC tumor cells

**Fig 1:**
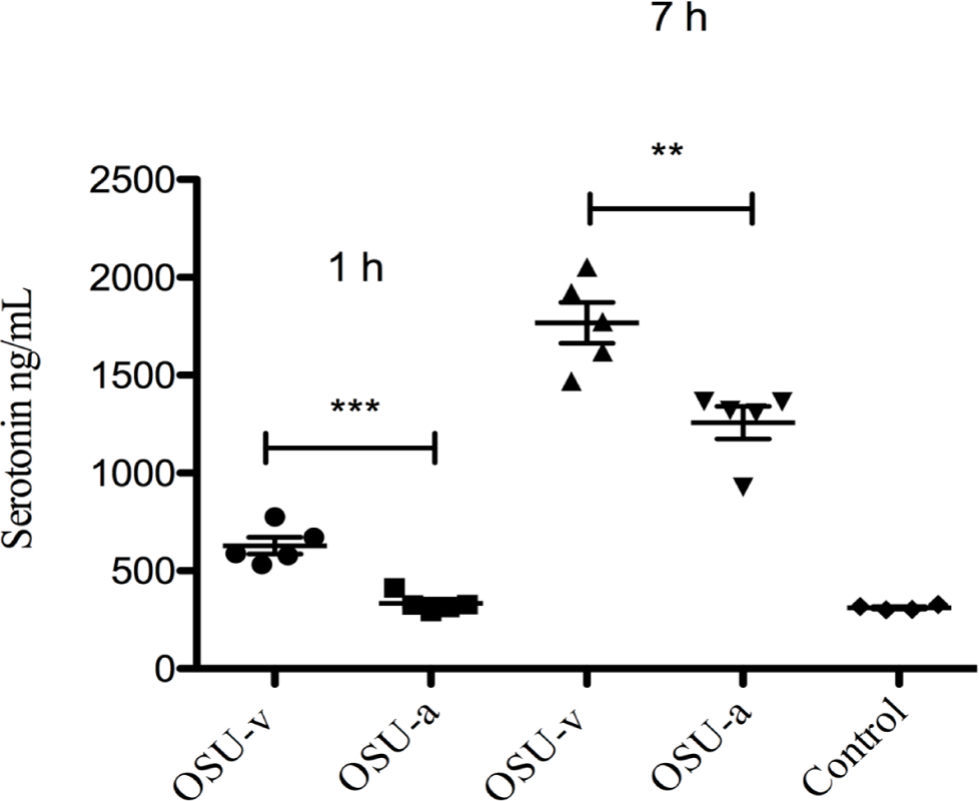
Virulent OSU virus stimulates more release of serotonin from EC tumor cells than attenuated OSU virus. Following infection (MOI = 1) of EC tumor cells with attenuated OSU-a and virulent OSU-v virus for 1 and 7 h p.i, media was collected and the serotonin release determined with ELISA. Control: cell media from MA104 cells. Data is presented as mean ± SEM. *** = p ˂ 0.001 and ** = p ˂ 0.01 with Student’s t-test; n = 5.

We have previously shown that RV can infect EC tumor cells *in vivo* and *in vitro* and stimulate release of serotonin [3]. As serotonin is a known secretogue [14, 56] we thus investigated if RV-stimulated release of serotonin is a contributing virulence factor. To address this question we investigated two porcine RV strains (OSU-v and OSU-a) having distinct differences in capacity to mobilize intracellular calcium and to induce diarrhoea in infant mice [42]. As shown in Fig 1, both viruses stimulated release of serotonin, but the virulent OSU-v stimulated significantly more than the tissue culture-adapted OSU-a, both after 1 h p.i (p < 0.001) and 7 h p.i (p < 0.01) infection. Furthermore it should be noted that attenuated OSU-a (1 h) and control (MA104 cells) had similar low effect (Fig 1). To ascertain that identical MOI was used for both viruses, careful viral titration was done on EC tumor cells (S1 Fig). S1 Fig also concludes that both viruses could replicate in EC tumor cells. To confirm that 7 passages of OSU-v in MA104 cells had not resulted in mutations during cultivation, the NSP4 gene of OSU-v was sequenced and compared to the original OSU-v strain [42], no changes in the amino acid sequence was found (S2 Fig).

### Serotonin secretion from human EC tumor cells is attenuated upon stimulation by supernatant from NSP4 silenced RV-infected MA104 cells

**Fig 2:**
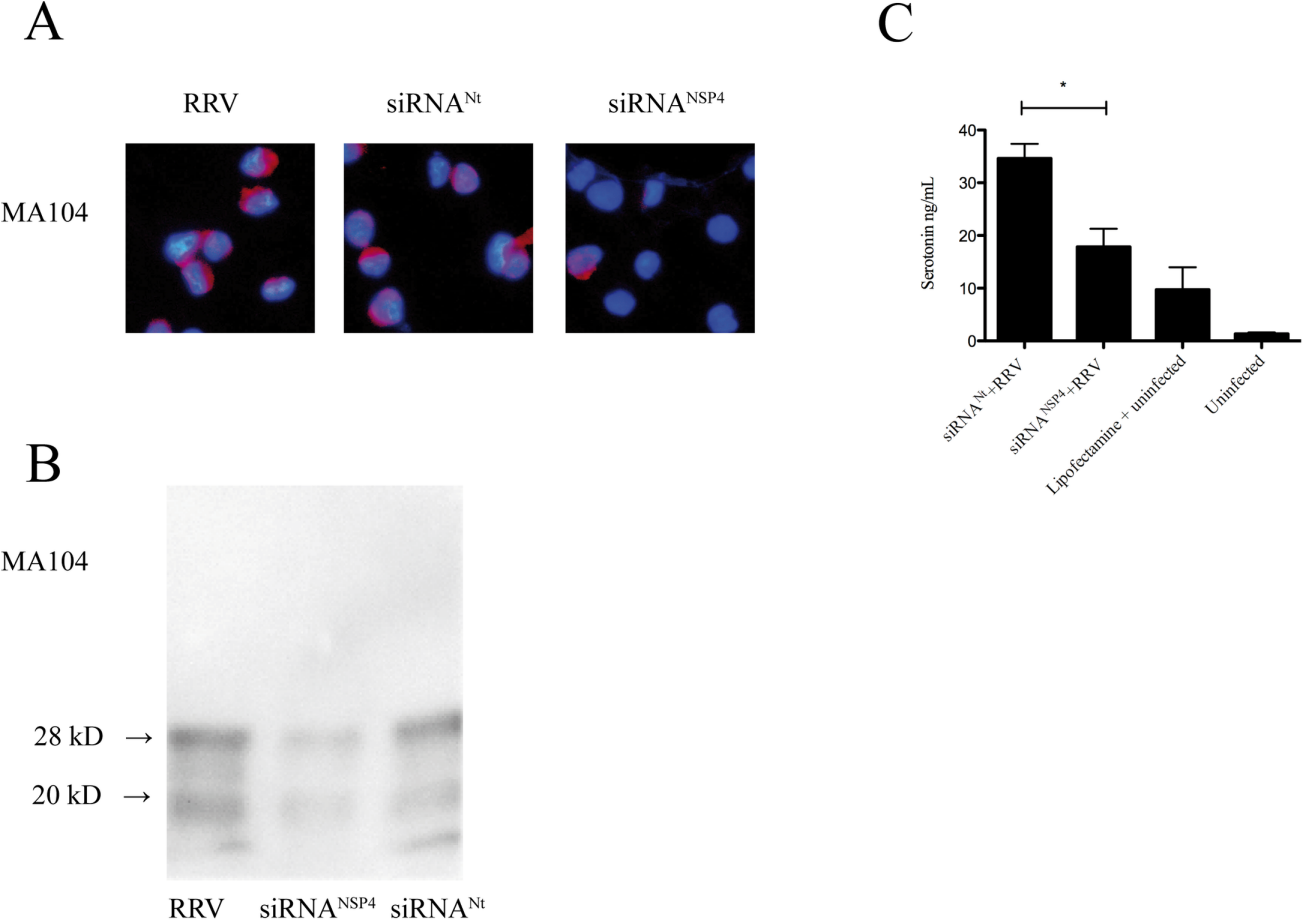
Extracellular stimulation of silenced NSP4 from MA104 cells attenuates secretion of serotonin from EC tumor cells. (A) MA104 cells were transfected with siRNA^NSP4^. At 24 h p.i cells were infected with RRV at a MOI of 0.5 and after 7 h p.i cells were harvested and stained for NSP4 by a rabbit anti- NSP4 and a rhodamine-conjugated goat anti-rabbit (red) conjugate. Nuclear staining was performed with DAPI (blue). (B) NSP4 western blotting of transfected and infected MA104 cells. Protein quantification performed and the relative protein concentration was determined. 28 kD = glycosylated form of NSP4, 20 kD = non-glycosylated form of NSP4. (C) EC tumor cells stimulated for 1 h with cell supernatants from MA104 infected for 7 h. Serotonin secretion was analysed by ELISA. Data is presented as means + SEM.* = p ˂ 0.05 with Mann-Whitney U test; n = 4. siRNA^Nt^ denotes non-targeting sequence.

We have previously shown that RV infection results in secretion of serotonin from human EC tumor cells *in vitro* and *ex vivo* [3]. To identify which viral protein(s) is (are) responsible for this unique property, VP4, VP6, VP7 and NSP4 gene expression were silenced. These are proteins with known biological functions and which previously have been successfully silenced [47–49]. In a first set of experiments we investigated whether these viral proteins had an extracellular effect on serotonin release from EC tumor cells. To test this, we first silenced the expression of NSP4, VP4, VP6 and VP7 in RRV-infected MA104 cells. After optimization, the efficiency of siRNA^NSP4^ silencing was approximately 66% in MA104 cells (Fig 2A and 2B) and 33% for siRNA^VP4^, 47% for siRNA^VP6^ and 35% for siRNA^VP7^. Supernatant was collected 7 h p.i and used to stimulate EC tumor cells. After 1 h, cell media was collected and serotonin content determined. As illustrated in Fig 2C, supernatant from MA104 cells silenced for NSP4 reduced secretion of serotonin from EC tumor cells with 48% as compared to siRNA^Nt^ (p < 0.05). In contrast no significant difference in serotonin secretion was observed from EC tumor cells stimulated for 1h with supernatant from VP4, VP6 and VP7 silenced and infected MA104 cells (S3 Fig).

### Intracellularly expressed NSP4 stimulates serotonin release from human EC tumor cells

**Fig 3:**
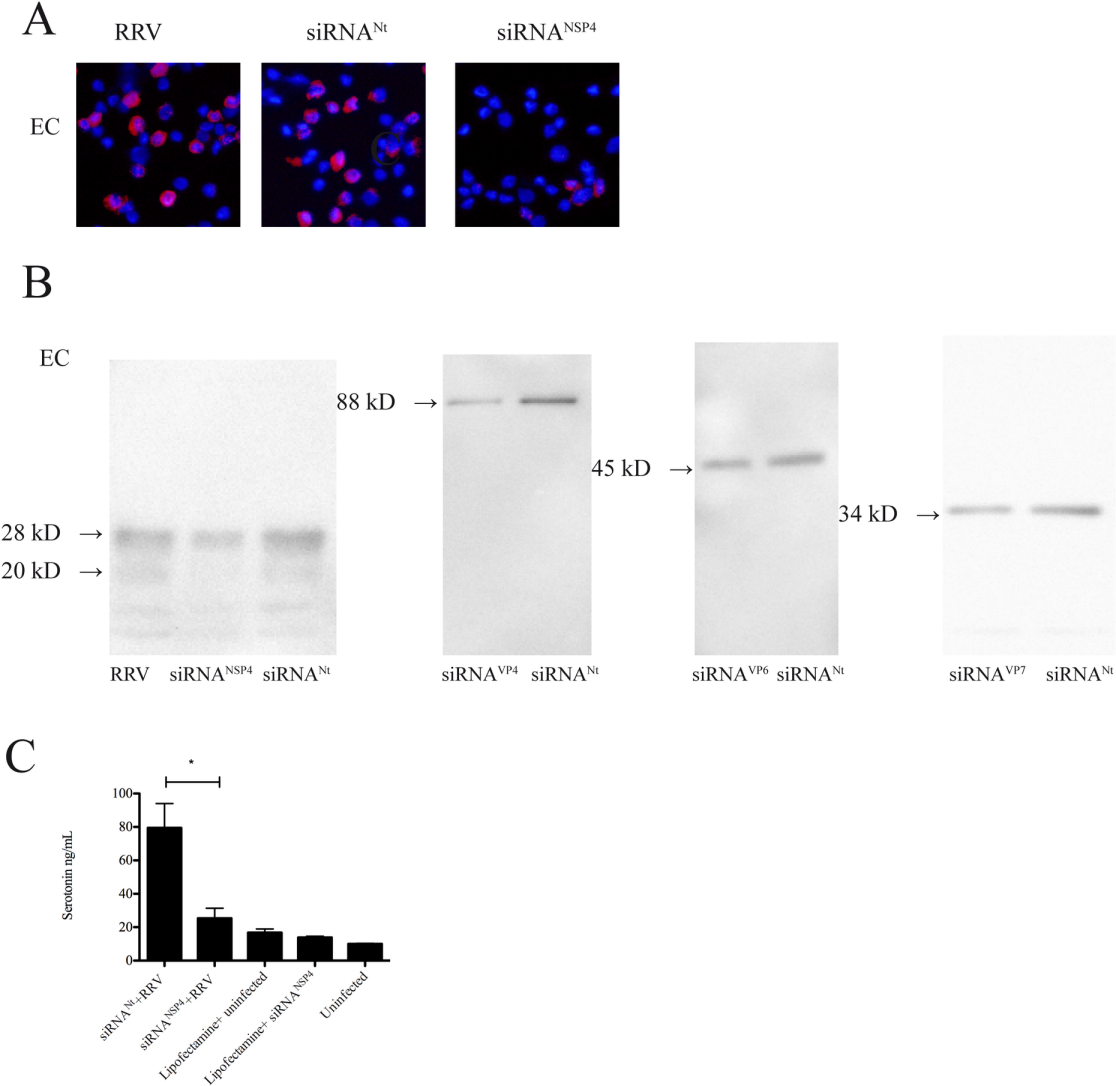
Intracellular expressed NSP4 stimulates serotonin release from human EC tumor cells. (A) EC tumor cells were transfected with siRNA^NSP4^. At 24 h p.i cells were infected with RRV at a MOI of 0.5 and after 7 h p.i cells were harvested and stained for NSP4 by a rabbit anti- NSP4 and a rhodamine-conjugated goat anti-rabbit (red) conjugate. (B) Silencing effect on NSP4, VP4, VP6 and VP7 expression in EC tumor cells. Following transfection and infection, cells were lysed and viral protein expression analysed by western blotting. With every western blot analysis, the amount of loaded protein was adjusted by comparing protein content in Comassie Blue-stained gels. 28 kD = glycosylated form of NSP4, 20 kD = non-glycosylated form of NSP4. (C) EC tumor cells transfected with siRNA^NSP4^ and infected. At 7 h p.i medium was changed and after 1 h supernatants were collected and analysed for serotonin. * = p ˂ 0.05 with Mann-Whitney U test; n = 4.

Next we investigated which of the intracellularly expressed viral proteins had effect on serotonin release from EC tumor cells. EC tumor cells were transfected with siRNA^Nt^ or siRNA^NSP4^/ siRNA^VP4^/ siRNA^VP6^/ siRNA^VP7^. After optimization, the efficiency of siRNA^NSP4^ silencing was approximately 63%, 52% for siRNA^VP6^, 81% for siRNA^VP4^ and 47% for siRNA^VP7^ (Fig 3A and 3B) in EC tumor cells. At 7 h p.i cell medium was replaced with new media and 60 min later media collected and analysed for serotonin content. As shown in Fig 3C, silencing of NSP4 attenuated secretion of serotonin from RRV-infected EC tumor cells by 68% compared to siRNA^Nt^ (p < 0.05). No statistic difference in serotonin secretion was observed from RRV-infected EC tumor cells silenced for VP4, VP6 and VP7 expression in comparison to infected cells transfected with siRNA^Nt^ (S4 Fig).

Basal secretion of serotonin was unaffected in uninfected EC tumor cells transfected with siRNA^NSP4^ compared to uninfected, non-transfected EC tumor cells, showing that siRNA itself did not affect serotonin secretion (Fig 3C). Furthermore, no effect of Lipofectamine *per se* was observed (Fig 3C). These results show that intracellularly expressed NSP4 has serotonin-stimulating properties.

### Rotavirus re-organizes serotonin appearance in human EC tumor cells

**Fig 4:**
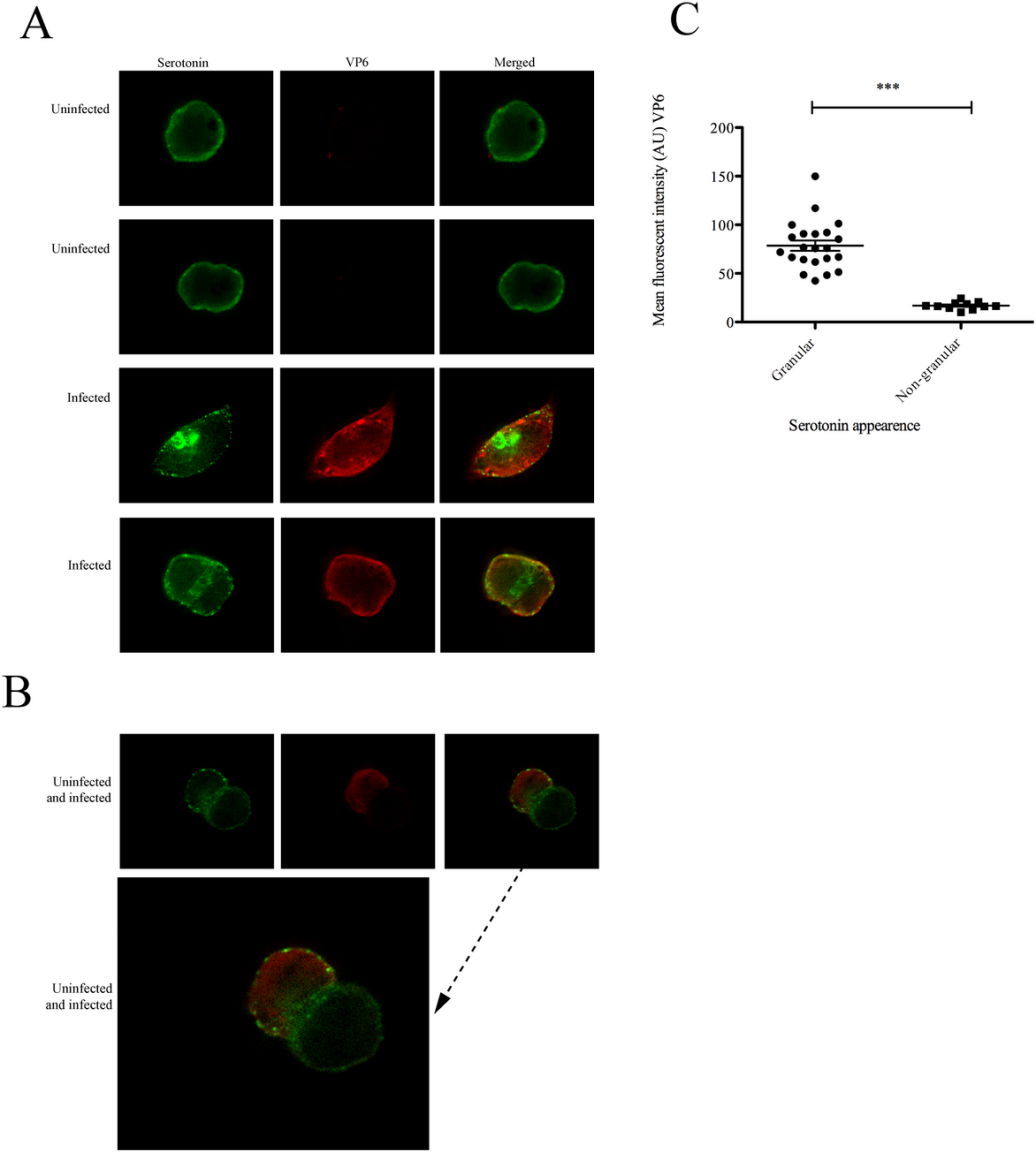
Rotavirus re-organizes serotonin appearance in EC tumor cells. (A) Infected and uninfected cells were co-stained for serotonin (green) and VP6 (red) expression. Infected cells displayed re-organization of serotonin from diffuse cytoplasmic and membrane-associated appearance in uninfected cells to an intense granular membrane-associated appearance in infected cells. (B) Illustration of an uninfected EC cell in close vicinity of an infected cell (red). Note the pronounced granular appearance of serotonin near the plasma membrane of the infected cell. (C) Fluorescent intensity (arbitar units, AU) (VP6) was calculated for each infected (n = 22) and uninfected (n = 13) cell and corresponding appearance (granular vs non-granular) taken into account. Data is presented as means ± SEM. *** = p < 0.001 with Mann-Whitney U test.

It is known that EC cells produce, store and release serotonin [10, 14] and that electron-dense serotonin granules upon specific stimulation are translocated to the cell membrane [57]. The fact that RV infection of EC tumor cells resulted in secretion of serotonin raised the question if infection *per se* would be associated with changes in appearance of serotonin-containing secretory granules. To address this question, the appearance and distribution of serotonin in RRV-infected *vs* uninfected EC tumor cells were investigated by confocal microscopy. At 18 h p.i RRV-infected EC tumor cells showed an intense granular membrane-associated appearance of serotonin compared to a rather diffuse pattern in uninfected EC tumor cells (Fig 4A and 4B). This particular appearance is illustrated in Fig 4B, with one infected cell in close vicinity to an uninfected cell. In addition, we observed a large number of granules of intense and thicker structures in the cytoplasm of RRV-infected EC tumor cells, as compared to uninfected cells, where such granules were almost absent. The appearance (intense large granular *vs* diffuse pattern) of serotonin was determined for each infected (n = 22) and uninfected (n = 13) cell and intense granular appearance of serotonin was significantly associated (p < 0.001) with infected EC tumor cells (Fig 4C).

### Rotavirus stimulation of serotonin from human EC tumor cells is calcium-dependent

**Fig 5:**
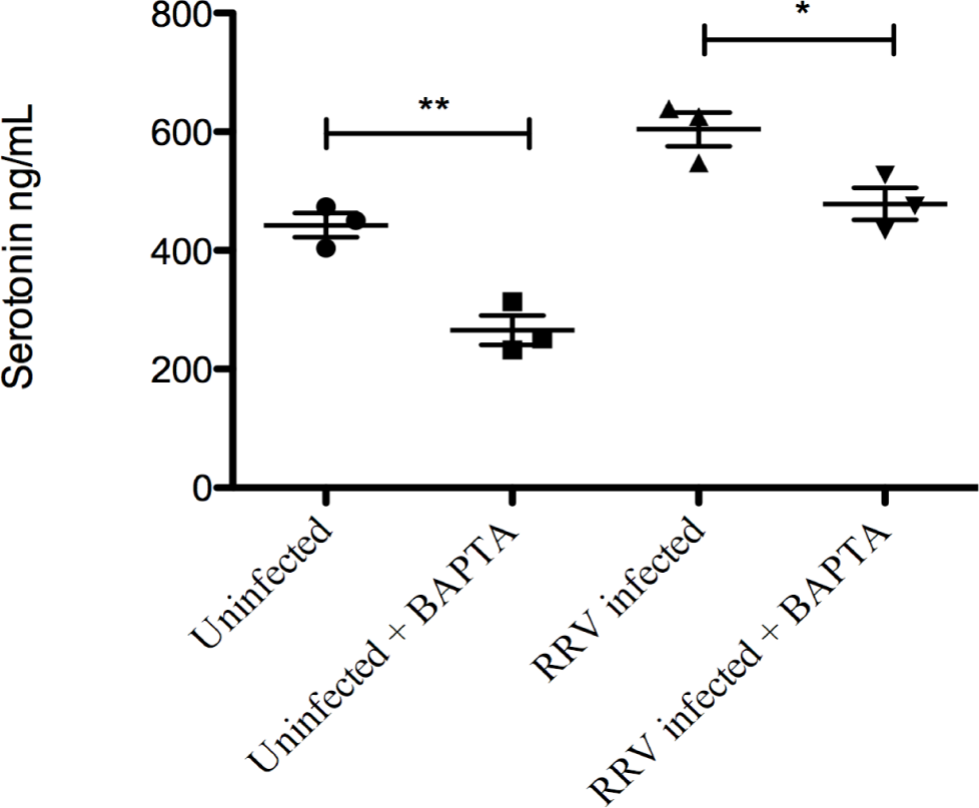
Release of serotonin from rotavirus-infected EC tumor cells is a calcium-dependent process. EC tumor cells were first infected with RRV (MOI = 0.5) and then at 3 h p.i BAPTA/AM was added to the cells to quench Ca2+ signals and 7 h p.i cell medium was collected for further serotonin ELISA assessment. Data is presented as means ± SEM. * = p ˂ 0.05 and ** = p ˂ 0.01 with Student’s t-test; n = 3.

Calcium is essential for proper RV maturation in the ER [58] and NSP4 has previously been shown to affect intracellular Ca^2+^ levels [59]. BAPTA/AM, a commonly used Ca^2+^ chelator [60, 61] was used to determine whether the release of serotonin from EC tumor cells was calcium-dependent. BAPTA/AM (25 uM) was added to RV-infected (MOI 0.5) EC tumor cells 3 h p.i. At 7 h p.i the supernatant was collected and serotonin content determined. As shown in Fig 5, secretion of serotonin from infected as well as from uninfected EC tumor cells were significantly attenuated (p ˂ 0.05 and p ˂ 0.01 respectively) by BABTA/AM.

### SERT mRNA expression is down-regulated in ileum of rotavirus-infected infant mice

**Fig 6:**
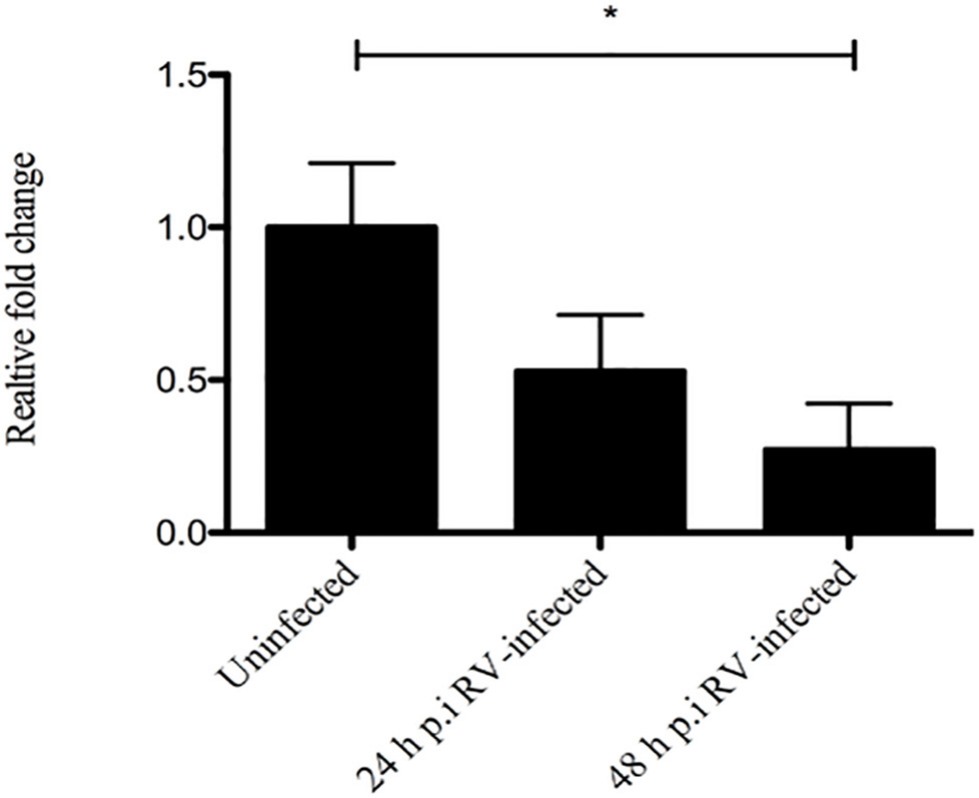
Rotavirus down-regulates SERT mRNA in ileum of infected mice. The small intestines of infected and uninfected mice were collected 24 and 48 h p.i and SERT and GAPDH mRNA levels quantified at each time-point by SYBR Green based real-time PCR. A significant down-regulation of SERT mRNA was found at 48 h p.i in ileum of infected compared to uninfected mice. Relative fold- change was set in relation to uninfected pups. Statistical analyses were done using Kruskal-Wallis and Bonferroni’s multiple comparison test. Data is presented as means + SEM. * = p < 0.05; n = 6.

The serotonin transporter protein SERT plays an important role in terminating and regulating the action of serotonin [30, 31]. It is thus possible that the magnitude of the nerve-stimulating signal by serotonin, released by EC cells, is serotonin-concentration dependent. Hence, we investigated the expression of *SERT* mRNA in RV-infected and compared to uninfected animals. In general, only low amounts of *SERT* mRNA were observed in duodenum and jejunum (real-time PCR Ct value > 35); thus, the differences were not possible to quantify, although the mRNA levels were generally lower in RV-infected mice. However, in ileum, *SERT* mRNA expression levels were higher and quantifiable and we observed a significant down-regulation of SERT expression in RV-infected mice (48 h p.i) compared to uninfected animals (average 3.7 fold, p < 0.05). A similar down-regulation of *SERT* mRNA was observed at 24 h p.i, although not statistically significant (Fig 6).

### TPH1 mRNA expression is unaffected in the small intestine of rotavirus-infected mice

**Fig 7:**
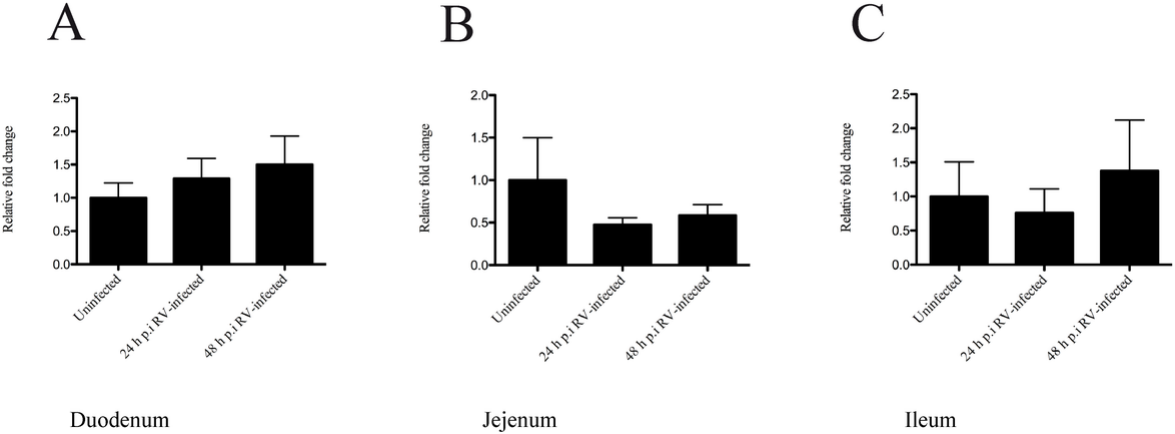
TPH1 mRNA levels are unaffected in the small intestine of rotavirus-infected pups. Infant mice were infected with EDIM (100DD50) and the small intestine collected 24 h and 48 h p.i followed by real-time PCR for TPH1 and GAPDH mRNA. No statistical difference in TPH1 mRNA levels compared to GAPDH mRNA was found in duodenum (A), jejenum (B) or ileum (C) of infected compared to uninfected pups. Relative fold-change is set in relation to uninfected pups. Statistical analyses were made using Kruskal-Wallis and Bonferroni’s multiple comparison test. Data is presented as mean + SEM; n = 6.

Serotonin is synthesized from L-tryptophan, and tryptophan hydroxylase (TPH1) catalyses the first step making it the rate-limiting enzyme in the biosynthesis of serotonin. TPH1 is known to be present in different cells including intestinal EC cells [28]. We expected to observe an up-regulation in *TPH1* mRNA levels in the *in vivo* model, as serotonin release is increased during RV infection *in vitro* [3]. However, there was a high variability in the mRNA levels among the animals and no significant differences in *TPH1* mRNA in duodenum, jejunum or ileum was observed in infant mice infected with EDIM (100DD_50_) compared to uninfected infant mice, neither at 24 or 48 h p.i (Fig 7A–7C).

### Serotonin induces rapid diarrhoea in infant but not in adult mice

Next we investigated if serotonin released from RV–infected EC tumor cells could stimulate diarrhoea in infant mice. Eight infant BALB/c mice were intra-peritoneally administered 50 μL cell supernatant from EC tumor cells containing 0.04 μg (7.4 μg/kg) of serotonin and 8 mice were given 2 x 0.04 μg. As control, 7 mice were administered supernatant from RRV-infected MA104 cells. Infant mice (n = 10) were also administered a higher dose (5 mg/kg) of serotonin. While none of the mice given supernatant from infected MA104 cells or EC tumor cells responded with diarrhoea, all mice (10/10) given the higher dose (5 mg/kg) of serotonin responded with diarrhoea within 30 min. It is well established that adult mice can be infected with RV, but do not respond with diarrhoea [62], presumably due to a block in water secretion from the small intestine [54]. To strengthen our proposed association between serotonin and RV-induced diarrhoea, adult BALB/c mice were intra-peritoneally administered 5 mg/kg of serotonin. None of the 8 adult mice responded with diarrhoea within 5 h. Thus similar as for RV these results demonstrate an age-dependent susceptibility of serotonin induced-diarrhoea in mice.

### Blocking of serotonin receptors attenuates rotavirus diarrhoea in infant mice

**Fig 8:**
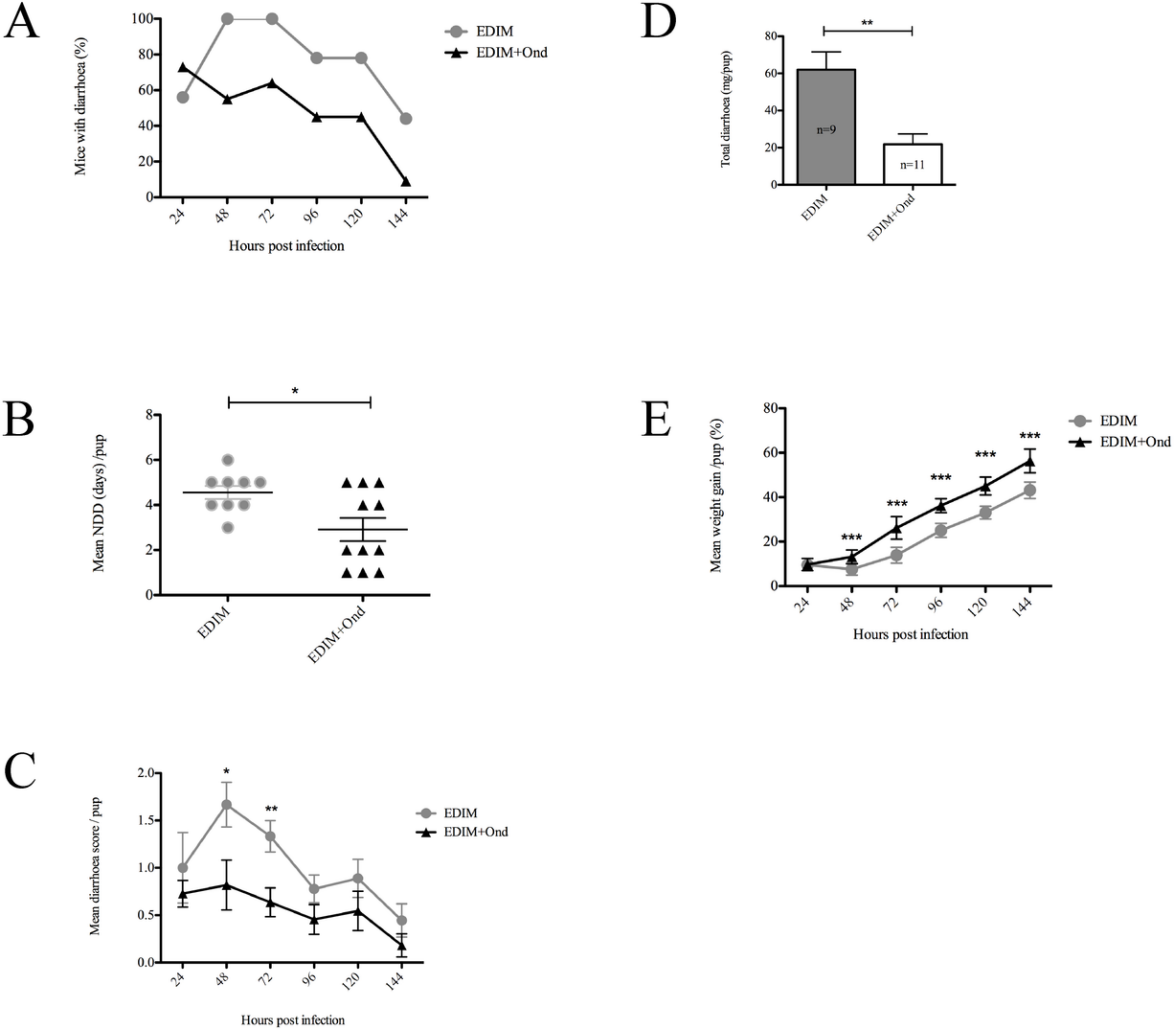
Serotonin antagonist Ondansetron attenuates diarrhoea in rotavirus-infected mice. (A) Prevalence of diarrhoea over time in rotavirus (EDIM) infected mice. Mice infected with EDIM were compared with mice infected with EDIM and treated with Ondansetron (5 mg/kg). Mean values are presented. (B) Number of days with diarrhoea (NDD) in mice infected with EDIM or EDIM plus Ondansetron (5 mg/kg). Data is presented as mean ± SEM. * = p < 0.05 with Student’s t-test. (C) Daily diarrhoea score for each day between the groups of mock-treated infected mice and infected mice treated with Ondansetron. Mock-treated infected mice received significantly more severe diarrhoea after 48 and 72 h p.i. Data is presented as mean ± SEM. * = p < 0.05 and ** = p < 0.01 with Student’s t-test. (D) Total amount (mg) of diarrhoea per mouse up to 144 h p.i. Data is presented as mean +SEM.** = p < 0.01 with Student’s t-test. (E) Comparison of weight gain between treated and mock-treated mice over time. Treated infected mice had gained significantly more weight from 48 h p.i. in comparison to only those without treatment. Data is presented as mean ± SEM. *** = p < 0.001 with Student’s t-test. EDIM (n = 9), EDIM + Ondansetron (n = 11).

To address our hypothesis that serotonin is associated with RV-induced diarrhoea, infant BALB/c mice were infected with wild type EDIM (100DD_50_) and orally treated with Ondansetron twice a day with 5 mg/kg. We used the serotonin-3 receptor antagonist Ondansetron, as this drug is commonly used to attenuate vomiting in children with acute gastroenteritis [21, 22] and have anti-diarrhoeal and obstipation properties [38–41]. Fig 8A shows that Ondansetron-treatment twice daily reduced the number of mice with diarrhoea, starting at 48 h p.i until 144 h p.i when the experiment was terminated. Previous studies [63] have shown that infant mice infected with EDIM have diarrhoea peak at 48 h p.i a time point when Ondansetron-treatment began to show effect. All infected mice in the Ondansetron-treated and mock-treated group had diarrhoea at any given time point. Fig 8B shows that the mean number of diarrhoea days per pup was significantly (p < 0.05) lower in the intervened group compared to mock-treated, reducing the days from a mean of 4.5 to 2.9 days in the intervened group. To obtain more detailed information about the anti-diarrhoeal effect of Ondansetron, the severity of diarrhoea was scored for each mouse and time-point. Fig 8C shows that there was a significant difference in the diarrhoea severity (score) between intervened (n = 11) and mock-treated (n = 9) mice at 48 (p < 0.05), and 72 h p.i (p < 0.01). Fig 8D shows that mock-treated mice had significantly (p < 0.01) more diarrhoea per mouse than Ondansetron-treated mice and moreover, mock-treated mice had significantly more total diarrhoea (mean 558.1 mg) output than intervened mice (mean 240.17 mg) (p < 0.01). Considering the positive effect of Ondansetron on duration and severity of diarrhoea, we also investigated whether Ondansetron-treatment could effect weight development during the course of infection. As shown in Fig 8E, Ondansetron-treated mice had significantly (p < 0.001) better weight gain during the course of infection compared to mock-treated mice. Ondansetron itself did not induce diarrhoea in uninfected mice (n = 4) thus concluding that the drug itself had no effect on described factors in Fig 8. Altogether, this suggests that RV infection of infant mice triggers serotonin signalling that affects duration, severity of diarrhoea and consequently weight gain and that duration and severity can be attenuated by the serotonin-3 receptor antagonist Ondansetron.

### Blocking of serotonin receptors attenuates murine rotavirus shedding in adult mice

**Fig 9:**
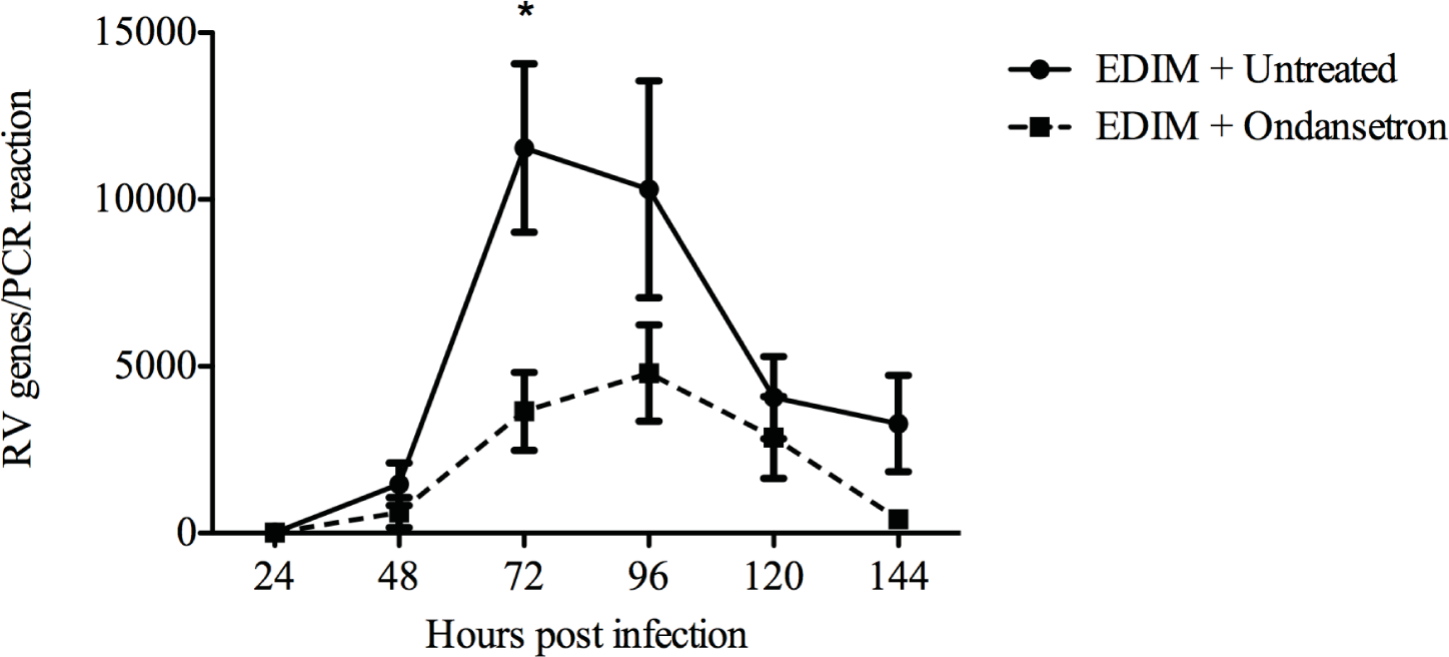
Ondansetron treatment reduces virus shedding. RV shedding as determined by real-time PCR in faeces of mice mock-treated or treated with Ondansetron. Mice were treated twice-a-day with Ondansetron (5 mg/kg). Total amount of RV shedding was significantly reduced in Ondansetron-treated mice with 30676 vs 12365 genome copies for mock-treated and Ondansetron-treated mice respectively. Data is presented as mean ± SEM. * = p < 0.05 with Mann-Witney U test; n = 10 in each group.

Next we investigated if interference in serotonin signalling by a serotonin receptor antagonist could effect viral shedding independently of diarrhoea. Adult RV BALB/c mice were first infected with EDIM (100DD_50_) and treated orally with Ondansetron (n = 10) (5 mg/kg) twice a day or mock-treated (n = 10) and followed for up to 144 h p.i. As shown in Fig 9, intervention by the serotonin receptor antagonist significantly attenuated viral shedding at any given time point, most pronounced at 72 h p.i (p < 0.05). Moreover, the total amount of viral shedding during the course of experiment was significantly lower (12365 vs 30676 RV genome copies) in Ondansetron-treated mice.

The fact that RV RNA shedding was significantly reduced in Ondansetron-treated and infected mice led us next to investigate if the reduced shedding was due to an antiviral effect. To address this question we investigated if Ondansetron had any *in vitro* effect on (i) number of infected cells, (ii) viral RNA synthesis or (iii) production of infectious virus. Briefly, MA104 cells were infected with Rhesus rotavirus (strain RRV) and at 1 h p.i media was replaced with serum-free media containing 10 μM Ondansetron (highest non-toxic concentration determined by trypan blue staining). To determine if Ondansetron affected the number of infected cells, cells were fixed at 16 h p.i and the number of infected cells determined by staining for the intracellular expressed non-structural NSP4 protein, by a rabbit anti NSP4 antibody [44]. The experiment revealed no difference in number of infected cells between Ondansetron-treated (10 μM) and mock-treated MA104 cells (148 infected cells per 1.04 cm^2^ in infected and treated cells vs 171 infected and mock-treated cells) Furthermore, no inhibitory effect of Ondansetron was observed on viral RNA synthesis by quantitative qPCR at 48 h p.i (S5 Fig). Next, we investigated if Ondansetron had any effect on progeny virus. Ondansetron-treated (10 μM) and mock-treated RRV infected (MOI 1) MA104 cells (n = 3) were freeze-thawed at 16 h p.i. and virus titres were determined. We found similar infectious titre among Ondansetron-treated (mean titre 4.1x10^5^ pfu/ml) and mock-treated (mean titre 4.8x10^5^ pfu/ml) cells, suggesting that Ondansetron did not have any detectable antiviral effect on rotavirus.

## Discussion

In this work it is shown that intracellularly expressed NSP4 is associated with the release of the neurotransmitter serotonin from human EC tumor cells. By employing virulent and attenuated porcine RV it is evident that virulence factors such as the capacities to induce diarrhoea in infant mice and to mobilize intracellular calcium [42] correlate with the ability to stimulate release of serotonin from human neuroendocrine cells. Ondansetron significantly attenuated murine RV diarrhoea in infant mice and that the treated mice gained weight better than mock-treated animals. A surprising finding is also that the serotonin receptor antagonist attenuated viral shedding. The later observation may support a novel therapeutic strategy for intervention of RV illness.

By inhibiting the expression of RV capsid proteins VP4, VP6, VP7 and the non-structural protein NSP4 by siRNA silencing, it was shown that intracellularly expressed NSP4 did stimulate release of serotonin, which is to the best of our knowledge the first intracellularly expressed viral protein with a neurotransmitter-stimulating property. Since the experiments did not include all RV proteins it cannot be excluded that other non-investigated proteins have similar properties. By employing previously described siRNA sequences and methodologies; NSP4 expression was silenced by 63% in EC tumor cells. This is similar to those of previous studies using other cell lines [48, 49, 64]. Expression of VP6 was reduced by approximately 52%, VP4 by 81% and VP7 47% in EC tumor cells. The silencing efficiency depend on a wide range of factors including cell lines and type of cells, siRNA concentration, incubation times and transfection agents being used.

Ca^2+^ signalling is known to play a key role in diverse cell functions and pathological mechanisms including RV infections and replication [60, 64–66]. There are also studies reporting changes in Ca^2+^ homeostasis coupled to the NSP4 enterotoxin [59, 61, 67, 68]. Using the cell-permeable Ca^2+^ chelator BAPTA/AM, we found that secretion of serotonin from EC tumor cells was Ca^2+^-dependent (Fig 5), an observation in line with the proposed model for serotonin-induced secretion from EC cells [57, 69].

It is well-known that EC cells synthesize serotonin through hydroxylation and decarboxylation of tryptophan in the cytoplasm of the EC cell [14]. The reason for studying EC cells in this work is that they form the largest enteroendocrine cell population in the small intestine and the only enteroendocrine cell that can synthesise serotonin. Upon stimulation, these cells are believed to account for the larger part of extracellularly released serotonin [11, 12]. Newly synthesized serotonin is subsequently transported into and stored in secretory granules by a vesicular monoamine transporter (VMAT) [70, 71] or SERT [16, 29, 34, 35] and/or degraded to maintain a normal extracellular serotonin level. Upon specific stimulation, these secretory granules are transported to the cell membrane and their content, including serotonin is released from the granules after exocytosis. In this way, extracellular serotonin may possibly reach lamina propria, where it can stimulate nerve terminals. The importance of serotonin in the GI lumen [72] is well documented and together with earlier findings on RV and serotonin [3] we hypothesized EC cells and serotonin to be the sensors during RV infection. First, we investigated, the distribution of serotonin following a RV-infection and compared it with uninfected cells. We found distinct differences with intense granular appearance in the periphery of the infected cells in contrast to more diffuse serotonin localization in uninfected cells (Fig 4A and 4B). Moreover the infected cells had intense, thick structures in the cytoplasm. Putatively, the infection of EC tumor cells did promote translocation of serotonin from the cytoplasm to secretory granules and further extracellular release via exocytosis [14], *in vivo* presumably to *lamina propria* and nerve endings of the ENS.

It has previously been shown that RV infection induces changes in cytoskeleton and cell morphology. Cytoskeleton modifications, are sensitive to changes in calcium concentration, which have been associated with the action of NSP4 [61], suggesting, but do not proving, that NSP4-induced changes in Ca^2+^ homeostasis may result in actin filament re-arrangement and thus altered appearance of the serotonin-containing secretory granules.

SERT regulates the bioavailability of serotonin [30, 31] and medical consequences of a deficient function may result in sensory (e.g. pain) and secretory (diarrhoea) symptoms. Although it has been shown previously that serotonin is released following RV infection of EC tumor cells [3], no information is yet available regarding the mRNA expression pattern of SERT during viral infections. In patients with IBS and ulcerative colitis [16, 19, 73] most studies have reported a decreased expression of SERT in the large intestine [16, 74, 75] and one [76] a small up-regulation of mRNA in the small intestine of IBS patients. In an animal model of colitis one study showed decreased mRNA levels of SERT [77], while in others there was an increase or no difference [73, 76]. Camilleri and co-workers [73] collected RNA from rectal and sigmoid colon mucosal biopsies in IBS patients and analysed *SERT* mRNA expression and found the levels to be normal. Hence, there are contradictory results on SERT expression in GI inflammatory diseases. Here we observed a significant down-regulation of *SERT* mRNA in the ileum of infected mice at 48 h p.i compared to uninfected mice (Fig 6). The modest down-regulation at 24 h p.i might reflect that the most pronounced diarrhoea and enteric lesions with the murine RV strain EDIM occurs at 48 h p.i [63]. The levels of SERT mRNA in duodenum and jejunum were generally down-regulated but too low to allow for any firm conclusions. The finding that the highest *SERT* mRNA expression in uninfected animals was found in ileum corroborates with a previous human study [31]. Furthermore, the down-regulation of SERT mRNA in ileum of infected animals might be due to a more preferential localization to this intestinal segment and as a consequence more extensive effects on host protein synthesis. A previous study showed indeed that during an infection the mouse ileum contained 100-fold more RV RNA than jejunum or duodenum [54].

A question is whether the increased serotonin secretion upon the RV infection of EC tumor cells was a result of an accumulation of pre-made serotonin in secretory granules, or of increased TPH1 transcription and translation. Serotonin is synthesized from L-tryptophan by tryptophan hydroxylase (TPH1) catalysis. TPH1 is known to be present in different cells, including intestinal EC cells [28] and one might assume that TPH1 expression would correlate with SERT expression. This is however not always the case, in one study there was no difference in the *TPH1* mRNA expression in duodenum of IBS patients [76], whereas the same type of patients demonstrated reduced expression of *TPH1* mRNA in the large intestine [16, 76]. Hence, contradictory results have been reported not only for SERT but also TPH1 expression in IBS and UC patients. Some of the discrepancies could however be related to the intestinal segment being investigated. Similar to Kerckhoffs and co-workers who observed no alternation in the *TPH1* mRNA expression in duodenum of IBS patients [76], we did not find any statistical difference for it in the small intestine of RV-infected mice (Fig 7A–7C). This can suggest that in either case the release of serotonin primarily occured from pre-made rather than from newly synthesised serotonin. This could also explain the rapid release of serotonin following a RV challenge.

Since serotonin was capable to induce rapid (< 30 min) diarrhoea in infant mice (10/10), we aimed to determine whether differences in viral virulence could be associated with capacity to stimulate release of serotonin from EC tumor cells. To address this question we compared the effects of virulent and avirulent porcine OSU viruses with known virulence differences in calcium mobilization and diarrhoea [42]. Not only can OSU-v virus mobilize more intracellular calcium and cause more diarrhoea in infant mice than OSU-a [42], but it also elicited stronger serotonin release from EC tumor cells than attenuated OSU-a virus (Fig 1). It is likely that Ca^2+^ mobilization is a key regulator of the secretion of serotonin, since clamping with BAPTA/AM, reduced both the basic and RV-induced release of serotonin (Fig 5), which corroborates previous findings [3].

Infant mice were treated with the serotonin receptor antagonist Ondansetron with the objective to further test our hypothesis that serotonin is participating is RV-induced diarrhoea. This receptor antagonist is rather frequently used to attenuate illness in children with acute gastroenteritis [21, 22], to attenuate diarrhoea in patients with inflammatory bowel disease [38, 39]. Moreover this drug has obstipation as a side effect [40, 41]. The mean number of diarrhoea days/ pup was significantly lower in the Ondansetron-treated group compared to mock-treated (2.9 vs 4.5 days) (Fig 8B). Moreover, (i) there was a significant difference in diarrhoea severity score between intervened and mock-treated mice at 48 and 72 h p.i, (ii) mock-treated mice had significantly more total diarrhoea output than intervened mice and (iii) Ondansetron-treatment resulted in better weight gain (Fig 8A–8E). The latter finding should be put in the light of observations that serotonin help control food intake [78, 79] and that feeding behaviour in fasting mice may be related to C-fos activity in the hypothalamus and brainstem [80]. It should also be noted that RV infection activates C-fos in CNS [3], which suggests a common signalling pathway that may result in less appetite during RV illness.

To assess whether Ondansetron could affect viral shedding independent of diarrhoea, adult mice were infected and non-or Ondansetron-treated and the viral load determined by real-time PCR. A most surprising effect was that the drug significantly attenuated total viral shedding (Fig 9). To better understand the unexpected effect of Ondansetron on viral shedding in mice, *in vitro* studies were performed. These studies revealed that Ondansetron neither had any effect on the number of infected cells, nor did it impair RV RNA synthesis at 48 h p.i up to concentrations that are toxic to cells (S5 Fig). Furthermore, no effect of Ondansetron was observed on viral progenies with mean (n = 3) titre of 4.1x10^5^ pfu/ml after Ondansetron treatment and 4.8x10^5^ pfu/ml after mock treatment (n = 3), thus it remains unresolved how Ondansetron attenuates RV shedding in mice.

It is interesting to mention that serotonin participate in immune activation and inflammation. It has been proposed that serotonin released from EC cells in response to stimuli, such as toxins and microbes, can act on innate immune cells such as macrophages and dendritic cells, to activate a proinflammatory cytokine response and thereby influence interaction between innate immune and adaptive immune cells [81, 82]. The presence of EC cells in contact or very close proximity to CD3^+^ and CD20^+^ lymphocytes [24] suggests existence of such an interaction between EC cells and immune cells.

Serotonin is a potent secretagogue of electrolytes and fluids in the intestines of all species studied, including humans [14, 56]. The serotonin-induced secretion mechanism is consistent with Cl^-^/HCO_3_^-^ secretion, which presumably occurs from crypt cells [83]. Pathways by which serotonin evokes intestinal secretion are likely both neuronal and non-neuronal and include at least activation of the serotonin-3 receptor and a neural reflex, which is tetrodotoxin sensitive [14]. Tetrodotoxin-sensitivity is most interesting, as it has been previously shown that RV-induced electrolyte and water secretion can be attenuated by tetrodotoxin [4].

In conclusion, we show that intracellularly expressed NSP4 carry serotonin neurotransmitter-stimulating properties and that serotonin participates in RV–induced diarrhoea, which can be attenuated by the commonly used serotonin-3 receptor antagonist Ondansetron. This new information can be added to the proposed gut-nerve-brain cross-talk axis in RV illness.

## Supporting Information

S1 Fig Viral titration of OSU-a and the virulent OSU-v P7 virus on EC tumor cells. EC tumor cells infected with attenuated OSU-a virus and the virulent OSU-v P7 virus and stained for VP6 expression to evaluate rates of infection and equal amount of cells infected. EC tumor cells were infected with MOI = 1 with respective viruses and 18 hours post infection fixed and stained with specific antibodies against VP6 (red expression), as described in the Material and Methods. Virus titration was previously performed on MA104 cells and the calculations were applied on EC tumor cells.
(PDF)

S2 Fig No changes in the amino acid sequence were found in the NSP4 gene between OSU-v and OSU-v P7. To exclude possibility that the NSP4 gene of OSU-v strain had mutated during the 7 passages in MA104 cells, sequencing of the NSP4 gene was performed, as described in Material and Methods.
(PDF)

S3 Fig No difference in serotonin secretion was observed from EC tumor cells stimulated for 1h with supernatant from VP4, VP6 and VP7 silenced and infected MA104 cells. EC tumor cells stimulated for 1 h with cell supernatants from silenced and infected MA104 cells. Serotonin secretion was analysed by ELISA. Data is presented as means + SEM with Mann-Whitney U test; n = 4. siRNA^Nt^ denotes non-targeting sequence and ns denotes not significant.
(PDF)

S4 Fig No difference in serotonin secretion was observed from RRV-infected EC tumor cells silenced for VP4, VP6 and VP7 expression in comparison to infected cells transfected with siRNA^Nt^. EC tumor cells transfected with siRNA^VP4^, siRNA^VP6^, siRNA^VP7^ and siRNA^Nt^ and infected with RRV. At 7 h p.i medium was changed and after 1 h serotonin secretion was analysed. Data is presented as means + SEM. Statistics were made using Mann-Whitney U test; n = 4. siRNA^Nt^ denotes non-targeting sequence and ns denotes not significant.
(PDF)

S5 Fig No difference in RV gene copy number was observed between RRV-infected Ondansetron-treated and mock-treated MA104 cells. Quantitfication of RV genes 48 h p.i as determined by real-time PCR in supernatants and cell lysates of RRV-infected Ondansetron-treated (10 μM) and mock-treated MA104 cells. Data is presented in a log2 scale with geometric mean values and 95% confidence interval. No significant differences between the groups; n = 3.
(PDF)
