# Supplementary figures and images for: Rotavirus and Serotonin Cross-Talk in Diarrhoea
Source: PLoS One. 2016 Jul 26;11(7):e0159660. doi: 10.1371/journal.pone.0159660 (PMC4961431; doi:10.1371/journal.pone.0159660)

OSU-a

OSU-v P7

Uninfected

4X/0.1

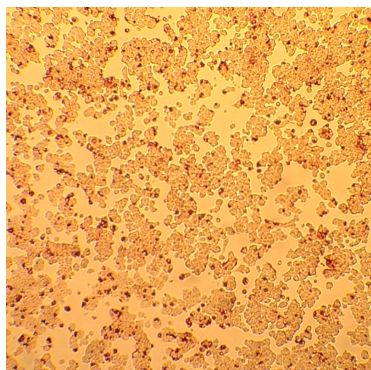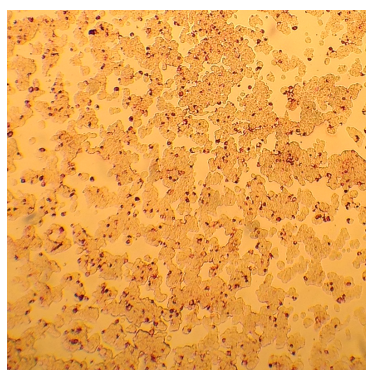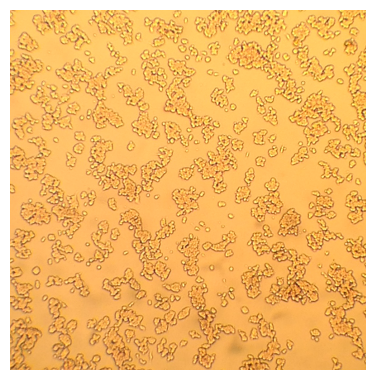

10X/0.22

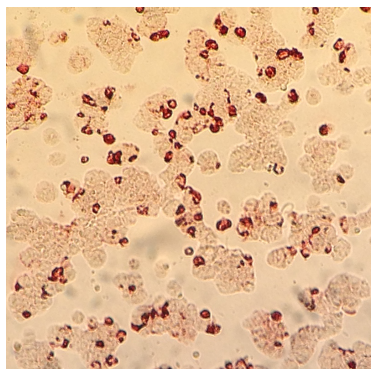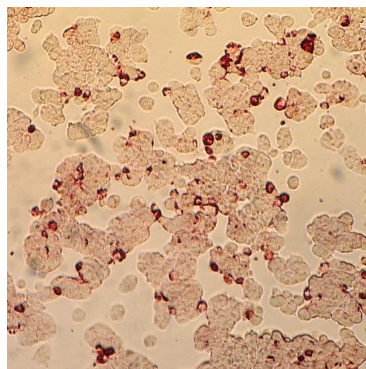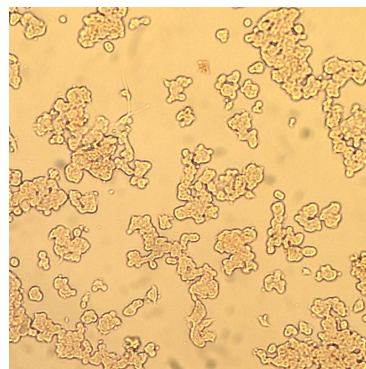

40X/0.5

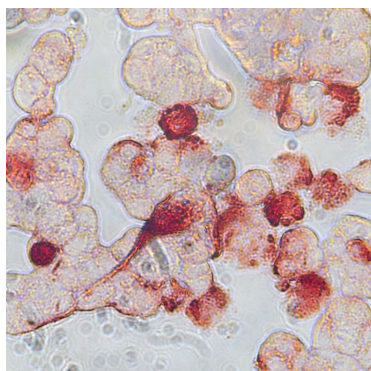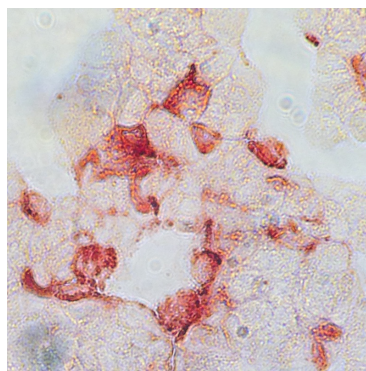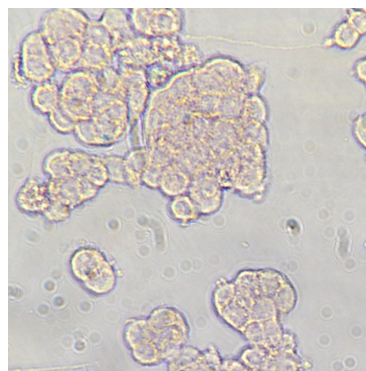

Supplement: S1 Fig — EC tumor cells infected with attenuated OSU-a virus and the virulent OSU-v P7 virus and stained for VP6 expression to evaluate rates of infection and equal amount of cells infected. EC tumor cells were infected with MOI = 1 with respective viruses and 18 hours post infection fixed and stained with specific antibodies against VP6 (red expression), as described in the Material and Methods. Virus titration was previously performed on MA104 cells and the calculations were applied on EC tumor cells. (PDF) [file pone.0159660.s001.pdf]

VP4

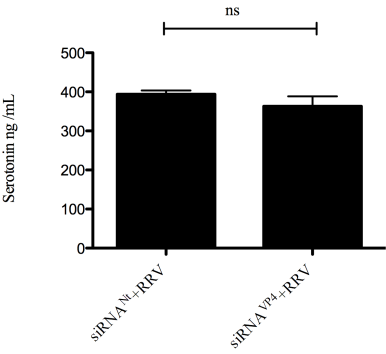

VP6

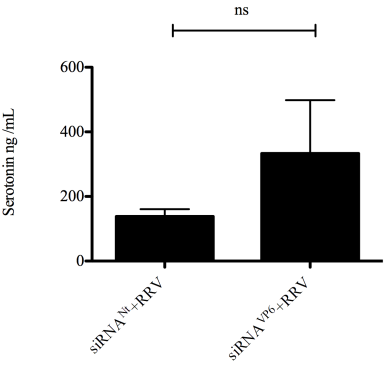

VP7

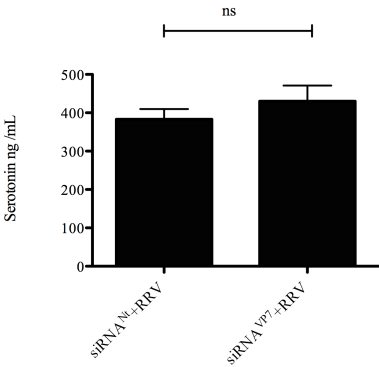

Supplement: S3 Fig — EC tumor cells stimulated for 1 h with cell supernatants from silenced and infected MA104 cells. Serotonin secretion was analysed by ELISA. Data is presented as means + SEM with Mann-Whitney U test; n = 4. siRNANt denotes non-targeting sequence and ns denotes not significant. (PDF) [file pone.0159660.s003.pdf]

VP4

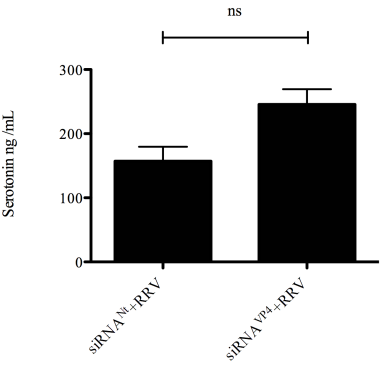

VP6

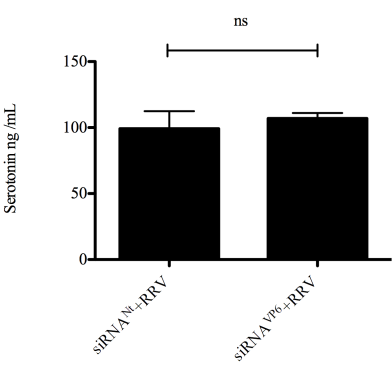

VP7

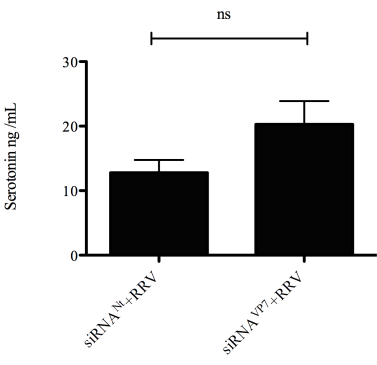

Supplement: S4 Fig — EC tumor cells transfected with siRNAVP4, siRNAVP6, siRNAVP7 and siRNANt and infected with RRV. At 7 h p.i medium was changed and after 1 h serotonin secretion was analysed. Data is presented as means + SEM. Statistics were made using Mann-Whitney U test; n = 4. siRNANt denotes non-targeting sequence and ns denotes not significant. (PDF) [file pone.0159660.s004.pdf]

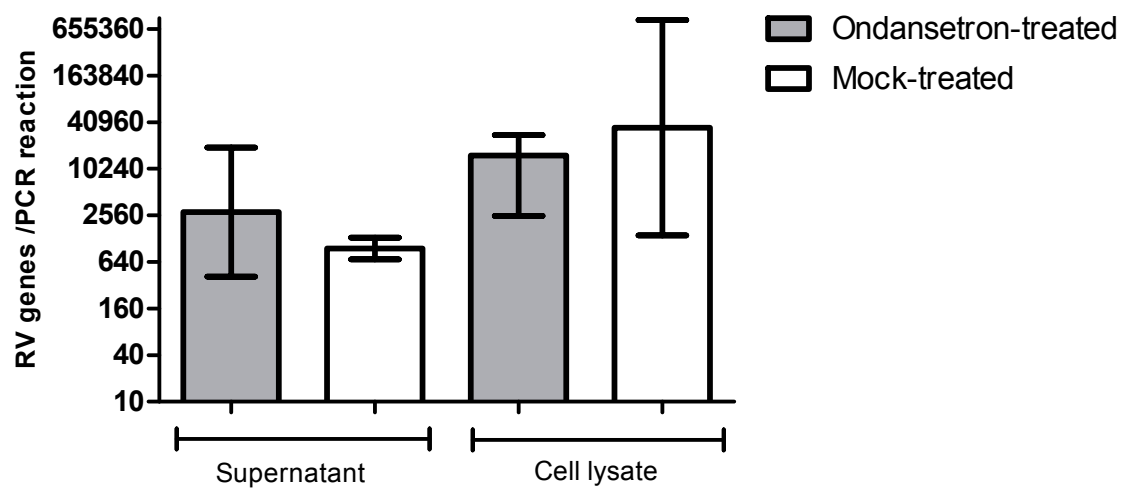

Supplement: S5 Fig — Quantitfication of RV genes 48 h p.i as determined by real-time PCR in supernatants and cell lysates of RRV-infected Ondansetron-treated (10 μM) and mock-treated MA104 cells. Data is presented in a log2 scale with geometric mean values and 95% confidence interval. No significant differences between the groups; n = 3. (PDF) [file pone.0159660.s005.pdf]
