# Supplementary material for: Rotavirus and Serotonin Cross-Talk in Diarrhoea
Source: PLoS One. 2016 Jul 26;11(7):e0159660. doi: 10.1371/journal.pone.0159660 (PMC4961431; doi:10.1371/journal.pone.0159660)
Supplement: S2 Fig — To exclude possibility that the NSP4 gene of OSU-v strain had mutated during the 7 passages in MA104 cells, sequencing of the NSP4 gene was performed, as described in Material and Methods. (PDF) [file pone.0159660.s002.pdf]

```
              17
OSU-v         MNDTLHSIIQDPGMAYFPYIASVLTVLFTLHKASIPTMKIALKTSKCSYKVIKYCMVTII
OSU-v P7      MNDTLHSIIQDPGMAYFPYIASVLTVLFTLHKASIPTMKIALKTSKCSYKVIKYCMVTII


OSU-v         NTLLKLAGYKEQVTTKDEIEQQMDRIIKEMRRQLEMIDKLTTREIEQVELLKRIHDKLAA
OSU-v P7      NTLLKLAGYKEQVTTKDEIEQQMDRIIKEMRRQLEMIDKLTTREIEQVELLKRIHDKLAA


OSU-v         RSVDAIDMSKEFNQKNIRTLDEWESGKNPYEPSEVTASM
OSU-v P7      RSVDAIDMSKEFNQKNIRTLDEWESGKNPYEPSEVTASM
                                                     175
```
